# The hidden potential of archaea in carbon and nitrogen cycling in agricultural soils: a review

**DOI:** 10.3389/fmicb.2026.1755559

**Published:** 2026-02-03

**Authors:** Brenda M. Speek, Afnan Khalil Ahmad Suleiman, Eline Keuning, Valentina Sechi, Cees J. N. Buisman, T. Martijn Bezemer

**Affiliations:** 1 Environmental Technology, Department of Agrotechnology and Food Sciences, Wageningen University, Wageningen, Netherlands; 2 Wetsus, European Centre of Excellence for Sustainable Water Technology, Leeuwarden, Netherlands; 3 Bioclear Earth B.V., Groningen, Netherlands; 4 Aboveground Belowground Interactions Group, Institute of Biology, Leiden University, Leiden, Netherlands

**Keywords:** ammonia-oxidizing archaea, haloarchaea, methanogenic archaea, n-damo archaea, nutrient cycling

## Abstract

The soil microbiome drives soil nutrient cycling and is intrinsically linked to plant productivity in agriculture. Archaea are members of many soil microbiomes and play important roles in nutrient cycling, particularly in the carbon and nitrogen cycle. Many archaeal groups contribute to both carbon and nitrogen cycles, but their dual roles are often underappreciated. For instance, ammonia-oxidizing archaea couple ammonia oxidation to carbon fixation, contributing to carbon sequestration in soils. Methanogenic archaea use ATP produced through methanogenesis for nitrogen fixation. N-DAMO archaea directly couple carbon and nitrogen cycling through nitrate-dependent anaerobic methane oxidation, while haloarchaea contribute to carbon sequestration and denitrification. Here, we synthesize the latest research regarding the dual roles of archaea in carbon and nitrogen cycling in agricultural soils. We pay special attention to how nutrient input influences these roles. We show that the relevance of the processes is highly context dependent. In addition, we identify several research directions that will help harness the difference roles of archaea in carbon and nitrogen cycling to increase agricultural productivity and sustainability. Finally, we showcase that abundance and activity of archaea in the soil microbiome could be steered through nutrient input or microbiome engineering strategies.

## 1 Introduction

Nutrient cycling is a central function of agricultural soils, maintaining fertility and supporting crop production (Jiao et al., 2019a). Because soil productivity directly affects food production, agricultural soils are typically closely managed (Tully and Ryals, 2017; Kopittke et al., 2019). Management strategies, such as nutrient input, directly impact biochemical nutrient cycling (Frink et al., 1999; Tully and Ryals, 2017). Understanding which processes drive nutrient cycling and how they increase soil productivity is therefore critical (Nealson and Stahl, 1997).

Key players in biochemical nutrient cycling are microbes (Sahu et al., 2017). These microorganisms contribute to maintain soil functionality and crop growth, by contributing to many different processes in soil nutrient cycling, including nitrification, nitrogen (N) fixation, carbon (C) turnover and phosphorus solubilization (Falkowski et al., 2008; Buée et al., 2009; Hartmann and Six, 2022). Bacteria are generally the most abundant group of microorganisms in soils (Bates et al., 2011; Wang et al., 2020). One bacterial species can perform multiple roles and thereby contribute to different nutrient cycles at the same time (Wang X. et al., 2024). Numerous studies have examined the roles of bacteria in agricultural soils; however, archaea have not received the same attention yet.

While previously considered extremophiles (Mehta and Baross, 2006; Dekas et al., 2009), this view has changed, as archaea have been found in common environments such as soils (Bates et al., 2011). Archaea are important members of the soil microbiome (Chen et al., 2008; Moissl-Eichinger et al., 2018; Naitam et al., 2023) and several archaeal species have been found to interact with plants (Chow et al., 2022). Specifically, archaea can improve plant growth through direct and indirect mechanisms; including contributing to biochemical nutrient cycling, mitigation of stresses in plants, phosphate solubilisation, siderophore production and degradation of pollutants (Odelade and Babalola, 2019; Alori et al., 2020; Singh et al., 2023; Martínez-Espinosa, 2024; Ventura et al., 2025). Particularly haloarchaea, known for their adaptation to saline environments, are frequently mentioned for their plant growth-promoting properties (Naitam et al., 2023), while many other archaeal species are known to play roles in biochemical nutrient cycling (Falkowski et al., 2008; Schleper and Nicol, 2010; Offre et al., 2013; Wright and Lehtovirta-Morley, 2023). The impact of archaea on plant growth and nutrient cycling underlines the importance of archaea in agricultural soils and plant productivity.

Agricultural soils can be divided into drylands, land used for crop growth and grasslands, and wetlands, including paddy fields (Jiao et al., 2019a). Dry- and wetlands typically harbour distinct archaeal communities due to differences in oxygen availability, irrigation and management practices (Jiao et al., 2019b). The most dominant archaeal phyla in soils are often the *Thaumarchaeota* and *Euryarchaeota*, recently reclassified as *Methanobacteriota* (Vaksmaa et al., 2016; Yuan et al., 2018; Clark et al., 2020; Megyes et al., 2021; Liu et al., 2022; Saghaï et al., 2022; Zhang et al., 2023). Both phyla have important roles in C and N turnover (Schleper and Nicol, 2010; Offre et al., 2013; Naitam and Kaushik, 2021).

The C- and N-cycles are essential in agriculture: they provide essential nutrients that support plant growth, but at the same time, can result in the release of several potent greenhouse gases (GHGs) (Shah et al., 2020). N plays an important role in plant development, and its deficiency can decrease plant growth, hinder root development and decrease plant dry mass (Frink et al., 1999). N application, in the form of chemical or organic fertilizer, is therefore essential for plants to obtain sufficient N (Ding et al., 2005; Diaz et al., 2006). Both bacteria and archaea play important roles in the transformation of N into ammonium (NH_4_^+^) and NO_3_^−^ (Cui et al., 2017), the primary sources of N for plants. Members of the *Thaumarchaeota*, specifically the ammonia-oxidizing archaea (AOA), are highly abundant in grasslands and crop lands, composing up to 95% of the total archaeal community (Clark et al., 2020; Megyes et al., 2021; Liu et al., 2022; Saghaï et al., 2022; Zhang et al., 2023). However, certain bacteria and archaea can transform nitrate (NO_3_^−^) into nitrous oxide (N_2_O), a GHG 273 times more potent than carbon dioxide (CO_2_), through denitrification (Spiertz, 2010; Hirsch and Mauchline, 2015).

The C-cycle influences key soil functions. Soil organic matter (SOM), which consists of approximately 58% organic C, is crucial for retaining soil structure and the water holding capacity of the soil (Manns et al., 2016). The soil microbiome is essential for the generation of SOM and plays a small role in sequestering atmospheric CO_2_ (Kallenbach et al., 2016; Jiang et al., 2022). Through microbial respiration, SOM is returned to the atmosphere as CO₂. Under anoxic conditions, methane (CH_4_), another potent GHG with a global warming potential 37 times greater than CO₂, is produced by methanogenic archaea (Derwent, 2020). However, methanotrophic archaea and bacteria can mitigate CH_4_ emissions by utilizing CH₄ for energy generation (McGlynn, 2017). Both methanogens and methanotrophs belong to the phylum of *Methanobacteriota. Methanobacteriota* thrive in anaerobic conditions, comprising more than 50% of the archaeal community in these environments (Vaksmaa et al., 2016; Yuan et al., 2018).

**Figure 1:**
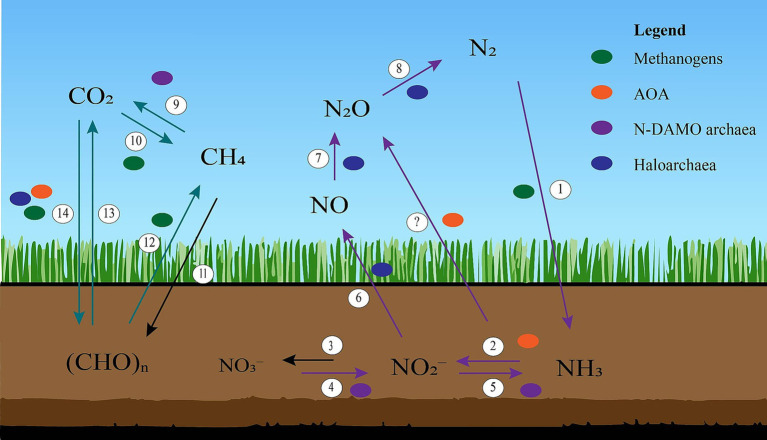
A simplified depiction of the carbon and nitrogen cycle in soil. 1) Biological Nitrogen Fixation (BNF), 2) Ammonia oxidation, 3) Nitrite oxidation, 4) Nitrate reduction, 5) Dissimilatory Nitrate Reduction to Ammonia (DNRA), 6) Nitrite reduction, 7) Nitric oxide reduction, 8) Nitrous oxide reduction, 9) Methane oxidation, 10) Methanogenesis, 11) Carbon assimilation, 12) Methanogenesis, 13) Respiration, and 14) Carbon fixation.

Above, four specific archaeal groups are mentioned: AOA, methanogens, methanotrophs and haloarchaea. These archaeal groups are typically characterized by a single well-known characteristic, i.e., ammonia (NH_3_) oxidation in AOA, CH_4_ production in methanogens and CH_4_ oxidation in methanotrophs, or salt tolerance and plant growth-promoting potential for haloarchaea. Yet, all four of these archaeal groups have the potential to contribute to both C- and N-cycling (Figure 1; Table 1). For example, AOA make contributions to N- and C- cycling through N_2_O formation and CO_2_ fixation (Pratscher et al., 2011). Methanogens contribute to N-cycling via N_2_ fixation (Leigh, 2000; Dekas et al., 2009). A specific subset of the anaerobic methanotrophs, N-DAMO archaea, contribute to NO_3_^−^ reduction and DNRA (Dissimilatory Nitrate Reduction to Ammonium) (Raghoebarsing et al., 2006; Wang et al., 2025). In contrast, while haloarchaea are not typically found in agricultural soils, they occupy a niche in saline environments, where they contribute to C turnover and denitrification (Torregrosa-Crespo et al., 2020a; Miralles-Robledillo et al., 2024).

**Table 1:** Overview of the relevant archaeal groups and their roles in C- and N-cycling.

Archaeal group	Role in C-cycling	Role in N-cycling	References
AOA	CO₂ fixation	Ammonia oxidation, N_2_O formation	Könneke et al. (2005), Francis et al. (2007), Pratscher et al. (2011), Jung et al. (2014a), Kobayashi et al. (2018), and Wan et al. (2023)
Haloarchaea	Photoheterotrophy, CO_2_ fixation	Denitrification	Grant (2001), Falb et al. (2008), Torregrosa-Crespo et al. (2020b), Torregrosa-Crespo et al. (2020a), and Miralles-Robledillo et al. (2021)
Methanogens	Methanogenesis, CO_2_ fixation	N₂ fixation	Reeve et al. (1997), Leigh (2000), Dekas et al. (2009), and Borrel et al. (2016)
N-DAMO archaea	CH_4_ oxidation	NO_3_^−^ reduction, DNRA, N_2_O formation	Raghoebarsing et al. (2006), Haroon et al. (2013), Nie et al. (2021), and Tan et al. (2024)

This review aims to increase the understanding of dual roles of AOA, methanogens, N-DAMO archaea, and haloarchaea in C- and N-cycling in agricultural soils. We highlight the potential ecological significance of their dual roles, with particular emphasis on their contributions to GHG emissions. By synthesizing current knowledge on the dual roles of these four archaeal groups in C- and N-cycling, we address the following questions: (1) Under which agricultural conditions are their roles in C- and N-cycling most relevant? (2) How does nutrient addition influence their activity and function? and (3) How can existing knowledge be used to improve current agricultural management practices or which future research is needed before current management practices can be improved? We aim to contribute to identifying research gaps and new research directions which will help in the development of agricultural strategies that utilize the underrecognized functions of archaea in C- and N-cycling to increase agricultural productivity and sustainability. To address these questions, we first provide an overview of the four archaeal groups that participate in both C- and N-cycling, highlighting their ecological niches and contributions to both nutrient cycles.

## 2 Archaeal groups with dual roles in C- and N-cycling

### 2.1 Ammonia-oxidizing archaea (AOA)

AOA catalyse the first and rate-limiting step of nitrification by oxidizing NH_3_ to nitrite (NO_2_^−^) (Könneke et al., 2005; Francis et al., 2007). AOA are often compared to ammonia-oxidizing bacteria (AOB), their bacterial counterparts that catalyse the same reaction. AOA and AOB occupy different ecological niches, but AOA can dominate in abundance under certain environmental conditions (Leininger et al., 2006; Sterngren et al., 2015; Mukhtar et al., 2019; Clark et al., 2020). All AOA fall into the class of *Nitrososphaeria*, within the phylum *Thermoproteota* (Rinke et al., 2021)*. Nitrososphaeria* are often the most dominant archaeal class in soils, composing over 70% of the total archaeal community (Megyes et al., 2021; Liu et al., 2022; Saghaï et al., 2022; W. Hu et al., 2022). The order *Nitrososphaeria* has been classified into several lineages with different characteristics based on NH_3_ affinity, optimum pH and urea usage, which allows the different lineages to occupy specific ecological niches (Lehtovirta-Morley et al., 2024; Zheng et al., 2024).

### 2.2 Haloarchaea

Haloarchaea thrive in saline and hypersaline environments (Oren, 2002; Baker et al., 2024). All haloarchaea belong to the class of *Halobacteria* within the phylum *Methanobacteriota* (Garrity et al., 2001). *Halobacteria* have been found to compose 46.5–89.5% of the total archaeal community in saline soils (Zhao S. et al., 2024). While typically not dominant in other soil types, they have been detected in drylands, where they can make up 20% of the archaeal community (Hu W. et al., 2022). Due to their reported plant growth–promoting properties, haloarchaea have been proposed as potential inoculants to enhance plant tolerance to different stressors (Yadav et al., 2015; Naitam et al., 2023; Mukhtar et al., 2024).

### 2.3 Methanogenic archaea

Methanogenic archaea are well-known for their exclusive contribution to CH_4_ production, as bacteria do not perform this process (Bapteste et al., 2005). While exceptions have been discovered, many methanogens belong to the phylum of *Methanobacteriota* (Wu et al., 2024). Due to their anoxic nature, methanogens are important contributors to C-cycling in waterlogged soil such as wetlands and paddy fields (Narrowe et al., 2019; Asakawa, 2021). Methanogenic archaea contribute up to 350–420 Tg of CH_4_ emissions per year (Lyu et al., 2018), with approximately 50% of CH_4_ emissions originating from wetlands and rice paddy fields (Lyu et al., 2018). Even though methanogens can play a prominent role in CH_4_ emissions in aerated soils (Angel et al., 2012; Angle et al., 2017), they have a low abundance compared to other archaeal groups in these environments (Angel et al., 2012; Alves et al., 2022).

### 2.4 N-DAMO archaea

As this review focuses on archaea that impact C- and N-cycling, here the focus will be specifically on archaea that perform nitrate-dependent anaerobic methane oxidation (N-DAMO). N-DAMO archaea belong to the genus of *Candidatus* (*Ca.*) Methanoperedens, within the phylum of *Methanobacteriota* (Raghoebarsing et al., 2006; Zehnle and Schoelmerich, 2025). N-DAMO archaea were first discovered in a consortium with N-DAMO bacteria, who use NO_2_^−^ for anaerobic oxidation of CH_4_, producing CO_2_ and nitrogen gas (N_2_) (Raghoebarsing et al., 2006). N-DAMO archaea reduce NO_3_^−^ during anaerobic CH_4_ oxidation, forming CO_2_ and NO_2_^−^ through reverse methanogenesis (Haroon et al., 2013; Wei et al., 2022). N-DAMO archaea are found in wetlands and paddy fields (Ding et al., 2016; Wang et al., 2022). In soils they are often detected in association with NO_2_^−^-utilizing bacteria who use the NO_2_^−^ produced by N-DAMO archaea (Ding et al., 2016). Many ANME (anaerobic methanotrophic archaea) have partnerships with sulphate reducing bacteria for electron shuttling, however, *Ca.* Methanoperedens does not need this symbiotic reaction (Zehnle and Schoelmerich, 2025).

## 3 Archaeal contribution to nitrogen cycling

### 3.1 Nitrification

AOA contribute to N-cycling in two major ways: (1) through NH_3_ oxidization, and (2) by producing N_2_O (Santoro et al., 2011). AOA and AOB perform oxidation of NH_3_ to hydroxylamine (NH_2_OH) using the enzyme ammonia-monooxygenase (AMO) (Zhalnina et al., 2012). In most AOA species, AMO exhibits a higher substrate affinity compared to AOB (Zhalnina et al., 2012). Under conditions with low N availability, AOA can therefore outcompete AOB (Ouyang et al., 2017). Moreover, AOA are known for their tolerance of acidic conditions (Gubry-Rangin et al., 2010; Yao et al., 2011; Zhalnina et al., 2012) and obligate acidophilic AOA species have been described (Lehtovirta-Morley et al., 2024). Under acidic conditions, NH₃ availability is reduced, leading to conditions that favour AOA (Prosser and Nicol, 2012). AOA are however not limited to acid soils and can grow in a wide pH range from acidic to alkaline (Prosser and Nicol, 2012). By contrast, AOB prefer soils at neutral pH (Robinson et al., 2014; Du et al., 2022). Finally, AOA have a higher affinity for oxygen than AOB, which can give them a growth advantage under oxygen-limited conditions (Du et al., 2022).

**Figure 2:**
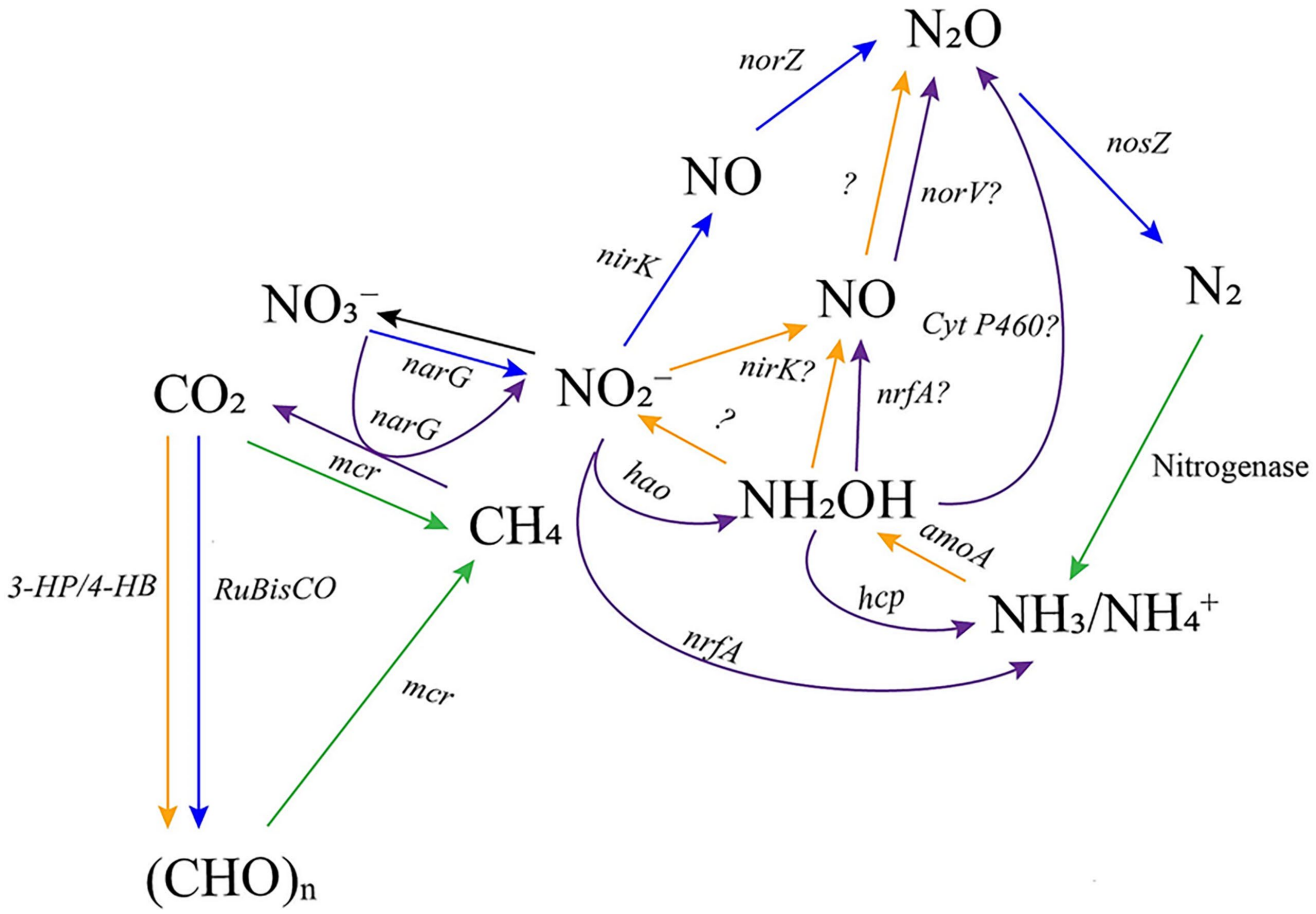
Key enzymes and simplified pathways involved in carbon and nitrogen cycling, specifically focused on the reactions mentioned in this review. The color of the arrow corresponds to the archaeal group involved in the reaction: Green = methanogens, Orange = AOA, purple = N-DAMO archaea, and blue = Halarchaea. Unidentified enzymes are indicated as “?”. NH_2_OH = hydroxylamine, NH_4_OH = ammonium hydroxide, 3-HP/4-HB = 3-hydroxypropionate/4-hydroxybutyrate (3-HP/4-HB) cycle, RuBisCO = ribulose-1,5-bisphosphate carboxylase/oxygenase, *amoA* = ammonia monooxygenase, *hao* = hydroxylamine oxidoreductase, *hcp* = hydroxylamine reductase, *nxr* = nitrite oxidoreductase (has not been identified in archaea), *nirS/nirK* = nitrite reductase, *norB* = nitric oxide reductase, *nosZ* = nitrous oxide reductase, *narG/narH* = nitrate reductase, *mcr* = methyl-coenzyme M reductase (involved in methanogenesis and reverse methanogenesis).

AMO has been identified as the enzyme catalysing the oxidation of NH_3_ to NH_2_OH (Vajrala et al., 2013). The process of NH_2_OH conversion to NO_2_^−^ is less well understood. AOB convert NH_2_OH to nitric oxide (NO) using hydroxylamine dehydrogenase (HAO), whereas another unidentified enzyme converts NH_2_OH to NO_2_^−^ (Caranto and Lancaster, 2017). AOA lack the gene encoding HAO, and this part of the NH_3_ oxidation process therefore remains unclear (Walker et al., 2010) (Figure 2).

Nevertheless, both groups can produce N_2_O during NH_3_ oxidation. In AOB, several routes of N_2_O formation have been proposed, including: 1) N_2_O formation via the oxidation of NH_2_OH to NO by HAO, 2) NO_2_^−^ reduction to NO via nitrite reductase (NIR) and NO reductase (NOR), and 3) oxidation of NH_2_OH by cytochrome P460 (Ritchie and Nicholas, 1972; Poth and Focht, 1985; Shaw et al., 2006; Caranto et al., 2016). Studies into the N_2_O production mechanism of marine AOA give several insights into the possible pathways of N_2_O production in AOA. Using isotope tracing experiments, Wan et al. (2023) found that AOA have several pathways of N_2_O production, depending on environmental conditions (Figure 2). Both NH_2_OH and NO were identified as key intermediates in AOA metabolism for N_2_O formation. Most notably, N_2_O formation was observed via NH_2_OH oxidation and reaction of NH_2_OH with NO. Under low oxygen conditions, *nirK* (copper-containing nitrite reductase) might be involved in the process of converting NO_2_^−^ to NO and O_2_ (Kraft et al., 2022; Hernández-Magaña and Kraft, 2024). O_2_ produced during the process can subsequently be used during NH_3_ oxidation. NO was further converted into N_2_O and N_2_ through the action of yet to be identified enzymes. *nirK* in AOA is likely involved in the generation of NO from both NO_2_^−^ and NH_2_OH, indicating several pathways for N_2_O production (Kobayashi et al., 2018). Jung et al. (2014b) studied the N_2_O production by five soil AOA. N_2_O production by soil AOA primarily took place via NH_2_OH oxidation and via nitrifier denitrification from NO_2_^−^, indicating that soil AOA also have different pathways for N_2_O production (Jung et al., 2014b).

In two marine AOA species, N_2_O production increased with decreasing oxygen concentrations (Qin et al., 2017). It is unclear if this is also the case for soil AOA. When the N_2_O production of *Nitrososphaera viennensis*, a soil AOA, *Nitrosopumilus maritimus*, a marine AOA, and *Nitrosospira multiformis,* an AOB, were compared, no increase in N_2_O production was observed at lower oxygen concentrations, while more N_2_O production was measured for the AOB species (Stieglmeier et al., 2014). Niche differentiation within the AOA can play a role in N_2_O emission by different AOA species (Jung et al., 2019). *Ca*. Nitrosotenuis chungbukensis from the order *Nitrosopumilales*, demonstrated higher yields of N_2_O than *Ca*. Nitrosocosmicus oleophilus from the order *Nitrososphaerales* (Jung et al., 2019). The amount of N_2_O produced seemed to depend on the environmental pH. At pH 7.5, N_2_O production originated largely from NH_2_OH. In acidic environments, *Ca*. N. oleophilus seems to increase N_2_O production. Interestingly, an isotope tracing experiment showed that *Ca.* N. oleophilus produced >50% of N_2_O from NO_2_^−^, which would suggest N_2_O formation through a nitrifier denitrification pathway (Jung et al., 2019). In addition, upregulation of a putative cythochrome P450 NO reductase was observed under acidic conditions. Cytochrome P450 is an enzyme involved in conversion of NO to N_2_O (Harris, 2002). This enzyme is also used for NO reduction by other microbial species (Higgins et al., 2018). These results suggest that *Ca.* N. chungbukensis and *Ca.* N. oleophilus use different pathways for N_2_O formation under acidic conditions. These findings exemplify the importance of studying the N_2_O yields of different AOA lineages.

### 3.2 Denitrification

Denitrification is the reduction of NO_3_^−^ to N_2_, via NO_2_^−^, NO and N_2_O (Bakken et al., 2012; Thomson et al., 2012). Some microorganisms possess all genes for denitrification and can carry out the entire pathway, whereas others lack the capacity to reduce NO_3_^−^ all the way to N_2_ and therefore perform partial denitrification (Babbin et al., 2015). Many haloarchaea are predicted to perform (partial) denitrification (Torregrosa-Crespo et al., 2018; Torregrosa-Crespo et al., 2019; Miralles-Robledillo et al., 2021). Haloarchaea likely perform denitrification through a similar pathway as denitrifying bacteria (Torregrosa-Crespo et al., 2020a; Miralles-Robledillo et al., 2024) (Figure 2). Some haloarchaea, such as *Haloferax mediterranei*, can reduce NO_3_^−^ all the way to N_2_, while others are predicted to catalyse partial denitrification potentially contributing to N_2_O formation (Torregrosa-Crespo et al., 2020b).

Four enzymes are involved in the reduction reactions of the denitrification pathway: membrane-bound nitrate reductase (NAR) or periplasmic nitrate reductase (NAP), NIR, NOR and nitrous oxide reductase (NOS) (Bothe et al., 2000). Many haloarchaea encode the *NarG* and *NarH* subunits of NAR (Miralles-Robledillo et al., 2021). The second step, NO_2_^−^ reduction, is performed by one of two main nitrite reductases: *nirS* (cytochrome-*cd1*-dependent nitrite reductases) or *nirK* (Kandeler et al., 2006). Genome analysis by Miralles-Robledillo et al. (2021) indicates that haloarchaea encode *nirK* and do not have *nirS*. In addition, the same study reveals that qNOR (*norZ*) is the main NOR in haloarchaea. Finally, haloarchaea encode *NosZ* for N_2_O reduction.

Considering the high relative abundance of haloarchaea in saline environments and the fact that half of the haloarchaea species are predicted to perform (partial) denitrification, they could significantly contribute to gaseous N-emissions (Shi et al., 2016; Torregrosa-Crespo et al., 2018; Miralles-Robledillo et al., 2021). Based on genome analysis it can be interfered which haloarchaea are complete denitrifiers and which are partial denitrifiers (Miralles-Robledillo et al., 2021). While denitrification by some species has been tested in laboratory environments (Torregrosa-Crespo et al., 2019) and RNA-seq studies are conducted to understand the denitrification process (Miralles-Robledillo et al., 2024), studies regarding their contribution to the release of N gasses in the environment are lacking.

### 3.3 Biological nitrogen fixation

N is most commonly present in the atmosphere in the form of N_2_, which is unavailable for most organisms (Erisman et al., 2008). N_2_ fixation, the reduction of N_2_ to NH_3,_ is carried out by a select group of bacteria and archaea known as diazotrophs (Boyd and Peters, 2013). Within the bacterial domain, N_2_ fixation is a more widespread ability, and N_2_-fixing bacteria can either be free-living, or plant associated (Pi et al., 2022). In the domain of archaea N_2_ fixation has, to date, only been demonstrated within methanogenic archaea (Leigh, 2000; Dekas et al., 2009). N_2_-fixing methanogens have been identified across several orders, including *Methanobacteriales*, *Methanosarcinales, Methanocellales* and *Methanomicrobiales* (Leigh, 2000; Koirala and Brözel, 2021). Notably, N_2_-fixing archaea are exclusively free-living (Pi et al., 2022). It is unclear whether N_2_ fixation first evolved in archaea and was subsequently transferred to bacteria or vice versa. Both the archaea-first and, more recently, the bacteria-first hypotheses have been proposed (Pi et al., 2022).

N_2_ fixation in methanogens is coupled to methanogenesis, as fixation requires the input of ATP generated through methanogenesis (Bae et al., 2018). N_2_ fixation is catalysed by the enzyme nitrogenase, of which three different variants exist (Riyaz and Khan, 2025): Fe-dependent, Vanadium (V)-dependent and molybdenum (Mo)-dependent nitrogenase. Mo-nitrogenase is the common type of nitrogenase, encoded by most, if not all, diazotrophs (Chanderban et al., 2023). In addition, it is the most efficient enzyme requiring the lowest amount of ATP for N_2_ fixation (Chanderban et al., 2023; Riyaz and Khan, 2025). Identified N_2_-fixing methanogens encode the Mo-dependent nitrogenase (Leigh, 2000). Some species, including *Methanosarcina acetivorans*, even encode all three variants (Chanderban et al., 2023). As Mo-nitrogenase is the common type of nitrogenase, N_2_-fixing microorganisms are often detected using *nifH*, encoding the Fe protein of Mo-nitrogenase, as a biomarker (Fani et al., 2000).

N_2_-fixing methanogens have been detected in a variety of different environments, including hydrothermal vents (Mehta and Baross, 2006), soil (Bae et al., 2018) and inside plant roots (Bao et al., 2025). Bae et al. (2018) used *nifH* to study activity of N_2_-fixating archaea in the Florida everglades, a freshwater wetland affected by the intensification of agricultural practices. They found that in soils unaffected by agricultural effluent, 49% of the *nifH* mRNA transcripts belong to methanogens. This indicates that there is a significant contribution of methanogenic archaea to N_2_ fixation in these wetlands.

Since N is an important element in agriculture, it is unsurprising that many studies have focussed on the effect of agricultural practices on diazotrophs. These studies use *nifH* as a biomarker for detection of both bacterial and archaeal diazotrophs (Fan et al., 2019; Han et al., 2019; Gao et al., 2021; Li et al., 2023; Zhou et al., 2023). However, the detection of N_2_-fixing archaea is often not reported in these studies. Because most samples were collected from the topsoil (0–20 cm), this may explain the absence of N₂-fixing archaea. If N_2_-fixing archaea were present, they are not further discussed, potentially due to their low abundance compared to N_2_-fixing bacteria (Nepel et al., 2022).

The use of *nifH* as a biomarker to detect archaeal N_2_-fixing methanogens has several limitations. Evaluation of different *nifH* primers revealed that not all primer pairs detect archaeal N_2_-fixers (Gaby and Buckley, 2012; Angel et al., 2018). Furthermore, many microbial species carry pseudo-*nifH*, they encode *nifH* but are unable to perform N_2_ fixation (Mise et al., 2021). This is the case for some anaerobic microorganisms, such as *Clostridia* species and some methanogens. Instead, other subunits of nitrogenase have been suggested as more suitable biomarkers, such as *nifD*, encoding the beta-subunit of nitrogenase (Mise et al., 2021). The occurrence of pseudo-*nifD* is less likely (Mise et al., 2021). Masuda et al. (2023) used *nifD* to determine the effect of fertilization on the N_2_-fixing community in paddy fields. They found a low abundance of total archaeal *nifD* compared to bacterial *nifD*, which indicates that bacteria are the major N_2_-fixers in these paddy fields. At genus level, the archaeal genus *Ca.* Methanoperendens was found amongst the top 5 of most abundant genera, particularly in unfertilized paddy fields (Masuda et al., 2023). The identification of *Ca.* Methanoperendens as a N_2_-fixer is surprising, as this archaeon is a methanotroph and not a methanogen. Since N_2_ fixation for archaea has only been identified in methanogens, the results of this study require further clarification and could potentially indicate that N_2_ fixation within archaea is not limited to methanogenic archaea. Evidence of possible N_2_ fixation by methanotrophic archaea has previously been found in methane seep sediments (Miyazaki et al., 2009).

### 3.4 Dissimilatory nitrate reduction to ammonium (DNRA)

N-DAMO archaea reduce NO_3_^−^ to NO_2_^−^ using electrons produced from CH_4_ via the reverse methanogenesis pathway (Haroon et al., 2013). This conversion is however not the only contribution N-DAMO archaea make to the N-cycle, as N-DAMO archaea can perform DNRA. In bacteria, DNRA is a two-step process where NO_3_^−^ is reduced to NO_2_^−^ and subsequently to NH_4_^+^ via NH_2_OH (Zhao et al., 2025). NO_3_^−^ is reduced to NO_2_^−^ by NAR or NAP and NO_2_^−^ is reduced to NH_4_^+^ by pentaheme cytochrome c nitrite reductase (*NrfA*) (Zhao et al., 2025).

N-DAMO archaea encode both *nrfA* and *narG*, indicating a similar pathway for NO_3_^−^ reduction as bacteria (Haroon et al., 2013; Nie et al., 2021). However, N-DAMO archaea seem to possess several different pathways for DNRA (Wang et al., 2025). Wang et al. (2025) discovered two possible pathways of DNRA in the presence of manganese (Mn): (1) ammonia-forming nitrite reductase (Nrf) pathway and (2) the reverse hydroxylamine:ubiquinone reductase module pathway. In addition to *nrfA*, *hao* and the gene encoding hydroxylamine reductase (*hcp*) were upregulated in the presence of Mn. This suggests that NO_2_^−^ was reduced to NH_2_OH by *hao* and subsequently to NH_4_^+^ by *hcp* (Figure 2).

Furthermore, N-DAMO archaea *Ca.* Methanoperedens nitroreducens was shown the perform DNRA in the presence of iron and low concentrations of NO_3_^−^ (Tan et al., 2024). Under the tested conditions, DNRA was coupled to N_2_O emissions. The gene encoding cytochrome P460 was upregulated, suggesting a role in catalysing the oxidation of NH_2_OH to N_2_O. Additionally, the nitric oxide reductase flavorubredoxin (*norV*) was upregulated, indicating the presence of a second N_2_O formation pathway, in which NO is produced by NrfA and subsequently reduced to N_2_O via NorV. The results of these studies suggest that DNRA may take place under specific conditions with different electron acceptors using different pathways.

## 4 Archaeal contribution to carbon cycling

### 4.1 Carbon fixation

AOA are generally considered to be obligate autotrophs, relying on atmospheric CO_2_ as their sole source of carbon through CO_2_ fixation. Pratscher et al. (2011) showed direct evidence of autotrophic growth in soil AOA using RNA-stable isotope probing. While a marine AOA species has been suggested to exhibit mixotrophic growth (Hallam et al., 2006), genomic analysis indicates that not all soil AOA are capable of mixotrophic growth (Lehtovirta-Morley et al., 2024).

CO_2_ fixation in AOA is coupled to NH_3_ oxidation (Pratscher et al., 2011). Without NH_3_ present, no CO_2_ fixation will take place. AOA fix CO_2_ via the 3-hydroxypropionate/4-hydroxybutyrate (3-HP/4-HB) cycle (Pratscher et al., 2011), this pathway is conserved within all AOA lineages (Lehtovirta-Morley et al., 2024). The gene encoding acetyl-CoA carboxylase *α*-submit (*accA*) is an essential gene involved in CO_2_ fixation in AOA (Yakimov et al., 2009). In contrast, AOB perform CO_2_ fixation via the Calvin-Benson-Bassham (CBB) cycle (Stein et al., 2007). A difference between the 3-HP/4-HB and CBB cycle is the energy required for CO_2_ fixation; the archaeal 3-HP/4-HP pathway requires less ATP then the CBB cycle (Könneke et al., 2014), making the archaeal pathway more efficient for biomass formation.

Most research regarding CO_2_ fixation by AOA and its environmental significance has been performed in oceans and deep seas. Due to the oligotrophic conditions in water, AOA make significant contributions to CO_2_ fixation (Wu et al., 2022). A study involving marine AOA and AOB found that *N. maritimus* formed 1.3 g dry biomass per mole NH_3_ oxidized, and the marine AOB *N. oceani* 0.8 g dry mass per mole (Könneke et al., 2014). This indicates that *N. maritimus* can form biomass more efficiently than *N. oceani*.

A study on AOA and AOB in forest soils estimated that AOA oxidize 59.8 μg N in the form of NH_3_ to fix 1 μg C to their biomass (Norman et al., 2015). For AOB this was 58.2 μg N (Norman et al., 2015). While this study looked at the whole community of AOA and AOB rather than single species, these results indicate that the efficiency of CO_2_ fixation is similar between both groups. To our knowledge there are currently no studies that performed similar experiments in agricultural soils.

Microcosm experiments with soils from grassland found that 80% of CO_2_ fixed into the SOC pool originated from AOA and AOB combined (Xia et al., 2022). Particularly in water amended soils without N addition, AOA contributed significantly to CO_2_ fixation, while AOB made large contributions under urea addition (Xia et al., 2022). This result is in line with the fact that AOA can outcompete AOB when N concentrations are low and may indicate that AOA might significantly contribute to CO_2_ fixation in N limited soils.

In contrast to AOA, Haloarchaea are considered photoheterotrophs, as they use organic C compounds as their C source and require light to generate energy (Grant, 2001). For example, *Haloarcula hispanica* was found to grow on acetate as a sole C source (Falb et al., 2008). However, comparative genomics has revealed exceptions: the haloarchaea *Natronomonas pharaonis* encodes an archaeal-type RuBisCO (ribulose-1,5-bisphosphate carboxylase/oxygenase), indicating that it might be capable of CO_2_ fixation (Falb et al., 2008). Whether other haloarchaea carry RuBisCO remains to be resolved.

Methanogens can also contribute to C-cycling via CO_2_ fixation. Specifically, autotrophic hydrogenotrophic methanogens who use CO_2_ and H_2_ as their sole source for C and energy generation (Borrel et al., 2016). Although heterotrophic hydrogenotrophic methanogens have been discovered as well (Kouzuma et al., 2017). Hydrogenotrophic methanogenic archaea are commonly detected in paddy fields, and several orders have been detected in grassland soils (Table 2).

**Table 2:** Methanogen groups capable of hydrogenotrophic methanogenesis and their habitats.

Order	Representative genera	Primary pathway of methanogenesis	Environment
*Methanobacteriales*	*Methanobacterium, Methanobrevibacter*	Hydrogenotrophic	Paddy soils (Zu et al., 2016; Yuan et al., 2018; Wang S. et al., 2023) and grassland (Hofmann et al., 2016)
*Methanomicrobiales*	*Methanoregula, Methanospirillum*	Hydrogenotrophic	Paddy soils (Vaksmaa et al., 2016; Zu et al., 2016; Yuan et al., 2018; Wang S. et al., 2023) and grassland (Hofmann et al., 2016)
*Methanocellales*	*Methanocella*	Hydrogenotrophic	Paddy soils (Vaksmaa et al., 2016; Zu et al., 2016; Yuan et al., 2018; Wang S. et al., 2023) and grassland (Hofmann et al., 2016)
*Methanosarcinales*	*Methanosarcina*	Methylotrophic, acetoclastic and hydrogenotrophic	Paddy soils (Zu et al., 2016; Yuan et al., 2018; Wang S. et al., 2023) and grassland (Hofmann et al., 2016)
*Methanococcales*	*Methanococcus*	Hydrogenotrophic	Grassland (Hofmann et al., 2016)

Most CO_2_ reducing methanogens fixate C via the reductive acetyl-CoA cycle, also known as the Wood-Ljungdahl pathway, but there are exceptions: *Methanospirillum hungatei* was found to encode RuBisCO and PRK (phosphoribulokinase), which are involved in the reductive hexulose-phosphate pathway for CO_2_ fixation (Kono et al., 2017; Lemaire et al., 2020). It is not clear if *M. hungatei* can grow with CO_2_ as a C source. For autotrophic hydrogenotrophic methanogens, CO_2_ is an important source for C and energy generation. A study by Chen et al. (2019) reports that *Methanobacterium congolense* converts four moles of CO_2_ into CH_4_ for each mole CO_2_ that was converted into biomass. This experiment included only one strain of *M. congolense* and was performed under CO_2_ limited conditions. Similar experiments could be conducted under ambient CO₂ concentrations and with other methanogen strains to determine whether rates are comparable. In soil environments where methanogens are abundant, CO_2_ fixation could potentially contribute to carbon sequestration. However, since CH_4_ has a higher global warming potential than CO_2_, emissions may outweigh the GHG reduction benefits of CO_2_ fixation (Danny Harvey, 1993).

### 4.2 Methanogenesis

Methanogenesis involves the degradation of organic matter into CO_2_ and CH_4_ (Conrad, 2020). While methanogenesis was initially thought to be a process solely occurring in anaerobic environments, it can also take place under aerobic conditions (Serrano-Silva et al., 2014; Angle et al., 2017). Methanogens rely on other members of the soil microbiome to obtain their substrates for methanogenesis (Conrad, 2020). Fermenting and hydrogenotrophic microorganisms degrade complex organic matter into CO_2_, H_2_ and simple organic compounds, which can in turn be utilized by methanogenic archaea (Conrad, 2020). Methanogens perform methanogenesis primarily through one of three pathways: (1) the methylotrophic pathway, (2) the acetoclastic pathway and (3) the hydrogenotrophic pathway (Liu and Whitman, 2008). Methylotrophic methanogenesis involves the utilization of methylated compounds as substrates (Liu and Whitman, 2008; Serrano-Silva et al., 2014). Acetoclastic methanogens use acetate as a substrate and hydrogenotrophic methanogens produce CH_4_ from CO_2_ and H_2_ (Liu and Whitman, 2008; Serrano-Silva et al., 2014). The hydrogenotrophic and acetoclastic pathways are the most common (Enrich-Prast et al., 2014; Kim and Whitman, 2014; Serrano-Silva et al., 2014). All methanogens encode Methyl-coenzyme M reductase (MCR), which is involved in the final step of methane formation (Enzmann et al., 2018). The mcr gene, particularly *mcrA*, is commonly employed as a molecular marker to detect methanogens in environmental samples. For a schematic overview of the three different pathways, we refer to Enzmann et al. (2018). For more in-depth information regarding enzymes involved in the methanogenesis process, we refer to the review by Reeve et al. (1997).

### 4.3 Nitrate-dependent anaerobic methane oxidation (N-DAMO)

CH_4_ oxidation is the process of oxidizing CH_4_ to CO_2_ (Serrano-Silva et al., 2014) (Figure 1). CH_4_ oxidation has been identified within both bacteria and archaea and can be performed under aerobic and anaerobic conditions (Wei et al., 2022; Zhao Y. et al., 2024). N-DAMO archaea perform CH_4_ oxidation anaerobically through the reduction of NO_3_^−^ to NO_2_^−^ (Raghoebarsing et al., 2006) (Figure 2). Anaerobic methane oxidation in archaea is thought to proceed via reverse methanogenesis, as N-DAMO archaea harbour enzymes used for CH_4_ formation in methanogens, including *mcr* (Hallam et al., 2004; Shima and Thauer, 2005; Haroon et al., 2013; Wei et al., 2022).

The N-DAMO process has been shown to contribute to 12–33% of total CH_4_ oxidation, N-DAMO archaea could therefore be promising for CH_4_ removal in different environments (Wang et al., 2022). In marsh soil, stable isotope probing was used to determine the contribution of N-DAMO archaea and bacteria to CH_4_ oxidation. The activity of N-DAMO bacteria was predicted to be 0.1–3.8 nmol ^13^CO_2_ g^−1^ dry soil day^−1^ and the activity of N-DAMO archaea 0.1 to 4.1 nmol ^13^CO_2_ g^−1^ dry soil day^−1^ (Zheng et al., 2020). Interestingly, a recent study showed that anaerobic CH_4_ oxidation can be an important source of SOM formation (Zhang et al., 2021). While this study did not distinguish between the different anaerobic CH_4_ oxidation pathways, approximately 60% of CH_4_ was turned into SOM instead of being respired as CO_2_.

## 5 The impact of nutrient input

Nutrient addition through fertilization is a common management strategy in agriculture, widely used to improve crop yield and quality by supplying essential nutrients, such as N, to soils (Bindraban et al., 2015; Ishfaq et al., 2023). Therefore, it is likely that the functions of the discussed archaeal groups in C- and N-cycling will be impacted by nutrient addition. Not only does nutrient input provide plants with nutrients for growth, the addition of nutrients can also increase emission of several GHGs.

Soil nitrification rates significantly increase with increasing N content in the soil (Li et al., 2020). It is therefore not surprising that fertilization will affect the activity of AOA in nutrient cycling. In particular, the type of fertilizer that is applied seems to be relevant, as growth and activity of AOA is increased under manure addition compared to synthetic fertilizer (Zeng et al., 2024; Wu et al., 2025). This is in line with the preference of AOA for lower levels of NH_3_, as manure releases NH_3_ slowly over time (Zhang et al., 2012; Prosser et al., 2020). Furthermore, long-term inorganic fertilizer application can reduce the pH of the soil, which can also lead to an increase in AOA (Ding et al., 2020). In addition, the N in fertilizer has the potential to increase the abundance of specific AOA lineages. In fields receiving manure with >900 kg/ha N, *Nitrosopumilus* was the predominant AOA lineage (Wang Z. et al., 2023). Application of manure with <600 kg/ha N led to higher abundance of *Nitrososphaera*. Even within the same AOA lineage, taxa can react differently to fertilizer input. For example, in acidic soil under different long-term organic and chemical fertilization regimes, *Nitrososphaerales* were the predominant AOA (Zhao et al., 2022). These regimes also led to differences in the abundance of *Nitrososphaerales* clades, likely driven by fertilizer-induced changes in soil pH. In paddy fields fertilized with manure, *Nitrosotalea devanaterra*-like AOA, from the *Ca. Nitrosotaleales* lineage, were the predominant AOA (Liu et al., 2018). The *Ca. Nitrosotaleales* lineage is known for its adaptation to acidic soils (Table 3), therefore its abundance was likely linked to the acidic pH in three out of the four studied soils.

**Table 3:** Characteristics of AOA lineages detected in soil environments based on cultured representatives.

AOA lineage^a^	Isolation source of representatives	Ammonia affinity	Capable of utilizing urea as a nitrogen source	Optimum pH range	N_2_O production (site preference value)	References
*Nitrosopumilales*	Marine water and agricultural soils	High (2.2 and 24.8 nM)	Yes^b^	7–8	pH 7.5: 29‰ pH 6.0: 27‰	Jung et al. (2019, 2022), Zhao et al. (2023), and Qin et al. (2024)
*Ca.* Nitrosotaleales	Acidic soil	Very high (0.6–2.8 nM)	No	5.3	Unknown	Jung et al. (2022) and Lehtovirta-Morley et al. (2024)
*Nitrososphaerales*	Agriculture soil, garden soil and thermal springs	Moderate to low (0.14 to 31.5 μM)	Yes	6.5–8.3	pH 7.5: 26‰ pH 5.5: 29‰	Jung et al. (2019, 2022) and Qin et al. (2024)
*Ca.* Nitrosomirales	East China Sea^c^	Unknown	Yes, based on metagenomics	7–8	Unknown	Zheng et al. (2024) and Li et al. (2025)

a The order of Ca. Nitrosocaldales contains thermophilic AOA species. The order appears to have limited relevance in soil environments and is consequently not mentioned (Luo et al., 2021).

b Not all strains encode urease (Zhao et al., 2023).

c Ca. Nitrosomirales is likely abundant in both marine and soil environments. However, contains only one cultured representative isolated from the East China Sea.

As nitrification rates increase, N_2_O emissions from AOA are also expected to increase. Several field studies have examined the contribution of AOA and AOB to N_2_O emission and the effects of nutrient input on the emission rates (Di et al., 2013; Hink et al., 2017; Hei et al., 2023; Zeng et al., 2024). In general, higher availability of NH_3_ and a neutral to alkalic pH are linked to increased contribution of AOB to nitrification (Rütting et al., 2021) and N_2_O emissions (Di et al., 2013; Hu L. et al., 2022; Zeng et al., 2024). Under N limiting conditions, AOA can exhibit a higher contribution to N_2_O emissions than AOB (Guo et al., 2025), likely because AOA are able to outcompete AOB at low substrate concentrations.

In line with this, the type of fertilizer applied to the soil might affect whether AOB or AOA produce more N_2_O. Hei et al. (2023) studied the effect of switching chemical fertilization to fertilization with manure in field plots. The N in the chemical fertilizer was applied in the form of urea and contained the same amount of N as the applied manure. Full substitution of urea with manure reduced N_2_O emissions from the plots (Hei et al., 2023). The reduction in N_2_O emissions correlated with a reduction in the abundance of AOB and an increase in the abundance of AOA. As the field plots received the same N concentrations, this reduction in AOB was most likely a result of the form of N used as fertilizer. It has been reported that AOB have higher affinity for urea than AOA (Qin et al., 2024), while manure releases N more gradually, favouring AOA (Zhang et al., 2012). It should be mentioned that the authors noted a 16–210% reduction of cumulative N_2_O emissions, and that cumulative emissions cannot be reduced more than 100%. Their observations are however supported by Hink et al. (2017), who found that AOA produce ∼0.5 × 10^−3^ N_2_O-N per NO_3_^−^-N produced and AOB ∼ 0.95 × 10^−3^ N_2_O-N per NO_3_^−^-N, suggesting that AOA produce less N_2_O during NH_3_ oxidation.

Likewise, CO_2_ fixation by AOA seems to be influenced by nutrient addition. Studies in soil often focus on the detection of CO_2_^−^ fixating microorganisms using qPCR and correlate gene abundance and CO_2_ fixation rates. *accA* has been used as an indicator gene for the 3-HP/4-HB pathway of AOA (Mao et al., 2024). In two different studies, one in a Mollisol with wheat-soybean-maize rotation and one in paddy soil, a positive correlation was found between *accA* abundance, autotrophic archaeal community composition and the CO_2_ fixation rate (Liao et al., 2020; Wang Q. et al., 2024). Specifically, in Mollisol, no fertilizer and manure treatment had higher *accA* copy numbers compared to NPK (nitrogen, phosphorus, and potassium) fertilizer and NPK fertilizer with manure (Liao et al., 2020).

In rice fields, conventional fertilization, consisting of NPK application, decreased the *accA* copy number, while it increased under conventional treatment combined with manure application (Wang Q. et al., 2024). The effect of manure combined with chemical fertilizer is therefore not in line with the results of Liao et al. (2020). This might indicate that fertilizer application is not the only regulator of the *accA* copy number. It is important, however, to mention that the gene abundance of the bacterial CO_2_ fixation pathway was much higher. Nevertheless, these findings indicate contribution of AOA to CO_2_ fixation in these soils.

Similar to AOA, it has been observed that methanogens react to fertilizer application and that different fertilizer treatments affect methanogen groups differently. Chemical and organic N application have been observed to increase CH_4_ fluxes (Kong et al., 2019). For example, acetoclastic methanogens increased in relative abundance during organic fertilization, while hydrogenotrophic methanogens decreased (Yuan et al., 2018). Acetoclastic methanogens require acetate as their substrate for methanogenesis, which is provided during fertilization with manure, but not during chemical fertilization. This observation was confirmed by Kong et al. (2019), who observed acetoclastic methanogens only under manure treatment.

The effect of management practices on CO_2_ and N_2_ fixation by methanogens is still an underexplored subject. The link between CO_2_ fixation and biomass formation has only been demonstrated in batch experiments for *Methanobacterium congolense* so far (Chen et al., 2019). In addition, the low abundance of N_2_-fixing methanogens in agriculture soils suggests that they do not play a major role in N_2_ fixation.

N-DAMO archaea require NO_3_^−^ for the oxidation of CH_4_. Hence, it is not surprising that they are affected by fertilizer input (Wang et al., 2022). The activity and abundance of N-DAMO archaea increases with fertilizer addition, and in turn, their increase in abundance leads to higher contributions to NO_3_^−^ and CH_4_ removal in paddy fields (Wang et al., 2022). In addition, the activity of N-DAMO archaea can potentially be optimized through supplementation of nutrients. Wang S. et al. (2024) demonstrated that through optimization of molybdenum, tungsten and selenium concentrations in growth media, the abundance of *mcrA* could be upregulated. Therefore, increasing the concentrations of these nutrients through fertilization might increase the CH_4_ oxidation rate of N-DAMO archaea.

Nutrient availability was observed to impact community composition of haloarchaea in saltwater samples (Hua et al., 2021). To our knowledge, no studies have been performed to determine the effect of nutrient input on this archaeal group. As haloarchaea are involved in denitrification, it can be speculated that the input of additional N can increase the emission of N gasses. With the potential application of haloarchaea as biofertilizers (Naitam et al., 2023), it will be essential to understand the contribution of haloarchaea to C- and N-cycling in the soil.

## 6 Detection of archaea in agricultural soils

The main challenge in archaeal research and uncovering their role in nutrient cycling, is the fact that many archaea have not been cultured in a laboratory setting (Sun et al., 2020; Rafiq et al., 2023). Several factors limit their culturability such as, lack of ability to mimic correct environmental conditions, and substrate concentrations and the slow growth rates that are typical for archaea. In addition, several archaeal species rely on other members of the soil microbiome for nutrient acquisition, which makes it extremely challenging to obtain pure cultures of these archaea and uncover their full contribution to nutrient cycling (Rafiq et al., 2023).

Despite the challenges in cultivating archaea, they can be studied in realistic environments such as agricultural soils. This is often done using several primers designed to target specific archaeal groups or functional genes (Table 4). For example, *amoA* primers are widely used for the detection of AOA and specific primers have been designed for the detection of *mcrA* from *M. nitroreducens*. Several *accA* primers have been used to target *accA* in AOA. *nifH* and *nifD* primers can be used for detecting N_2_-fixing methanogens, but these primers have not been specifically designed for the detection of N_2_-fixing methanogens.

**Table 4:** Collection of primers used for detection of archaea in literature cited in this review.

Target group	Gene	Primer name	Nucleotide sequence (5′-3′)	Used in reference	Notes^a^
Archaea	16S rRNA V3-V5 region	ARC344F	ACGGGGYGCAGCAGGCGCGA	Clark et al. (2020) and Hu W. et al. (2022)	
		ARC915R	GTGCTCCCCCGCCAATTCCT		
Archaea	16S rRNA V3-V4 region	S-D-Arch-0349-a-S-17	GYG CAS CAG KCG MGA AW	Saghaï et al. (2022)	
		S-D-Bact-0785-a-A-21	GACTACHVGGGTATCTAATCC		
Archaea	16S rRNA V3-V4 region	A519F	CAGCMGCCGCGGTAA	Megyes et al. (2021) and Liu et al. (2022)	
		Arch855R	TCCCCCGCCAATTCCTTTAA		
Archaea	16S rRNA	A109f	ACKGCTCAGTAACACGT	Angel et al. (2012)	
		A334r	TCGCGCCTGCTGCTCCCCGT		
*Thaumarchaeata*	*AmoA*	CrenamoA23f	ATGGTCTGGCTWAGACG	Hink et al. (2017), Clark et al. (2020), Hei et al. (2023), and Zeng et al. (2024)	
		CrenamoA616r	GCCATCCATCTGTATGTCCA		
AOA	16S rRNA	AP422F	GTCTAAAGGGTCTGTAGCCG	Mukhtar et al. (2019)	Primers were verified using the 16S ribosomal RNA gene of Nitrososphaera and Nitrosopumilus (Mukhtar et al., 2019).
		AP599R	TTCTGGTGAGACGCCTTCG		
*Thaumarchaeata*	*accA*	Crena529F	GCWATGACWGAYTTTGTY RTAATG	Liao et al. (2020)	
		Crena981R	TGGWTKRYTTGCAAYTATWCC		
Methanogens	Universal *mcrA*	mlas-mod – F	GGYGGTGTMGGDTTCACMCARTA	Angel et al. (2012)	
		mcrA-rev-R	CGTTCATBGCGTAGTTVGGRTAGT		
Diazotrophs	*nifH*	nifH-F	AAA GGY GGW ATC GGY AAR TCC ACC AC	Fan et al. (2019), Li et al. (2023), and Zhou et al. (2023)	Designed for the detection of bacterial diazotrophs (Rösch et al., 2002).
		nifH-R	TTG TTS GCS GCR TAC ATS GCC ATC AT		
Diazotrophs	*nifH*	PolF	TGCGAYCCSAARGCBGACT C	Han et al. (2019) and Gao et al. (2021)	Designed for the detection of bacterial diazotrophs (Poly et al., 2001). Gaby and Buckley (2012) found low coverage of this primer pair for cluster III sequences, which includes nifH sequences from N_2_-fixing archaea.
		PolR	CCATCATYTCRCCGGA		
Diazotrophs	*nifH*	IGK3	GCI WTH TAY GGI AAR GGI GGI ATH GGI AA	Nepel et al. (2022)	This primer pair was designed for the detection of bacterial N_2_-fixers (Ando et al., 2005). Gaby and Buckley (2012) found high coverage of this primer pair for cluster III sequences. Potentially produces high numbers of unspecific reads in soils (Angel et al., 2018).
		DVV	ATI GCR AAI CCI CCR CAI ACI ACR TC		
Diazotrophs	*nifD*	Universal nifD F	TGGGGICCIRTIAARGAYATG	Masuda et al. (2023)	Designed for the detection of bacterial nifD (Stoltzfus et al., 1997).
		Universal nifD R	TCRTTIGCIATRTGRTGICC		
M. nitroreducens-like archaea	*mcrA*	McrA159F	AAAGTGCGGAGCAGCAATCAC	Shen et al. (2021) and Wang et al. (2022)	
		McrA345R	TCGTCCCATTCCTGCTGCATTGC		
Cluster ANME-2d archaea	16S rRNA	DP397F	TGGCTGTCCAGCTRTYC	Ding et al. (2015)	Variations in amplification efficiency were reported (Ding et al., 2015).
		DP569R	GRACGCCTGACGATTRAG		

a Unless otherwise noted in this column, the amplification bias of the primer pair in soil environments has not been determined.

Specific primers for haloarchaea exist. For example, Oxley et al. (2010) designed primers for the detection of haloarchaea in human intestinal tracts. However, such primers have not been tested on soil samples as far as we are aware. While ample primers have been designed for the detection of bacteria involved in denitrification, these primers are not always efficient in detecting archaeal targets due to high sequence divergence (Ma et al., 2019). Specific primers have been designed to detect archaeal denitrification genes (Rusch, 2013), however to our knowledge these primers have not been tested using soil samples.

## 7 Concluding remarks and perspectives

With the involvement of archaea in both C- and N-cycling, archaea can make a significant contribution to the soil nutrient pool. Most archaeal groups are known to perform a specific role, but current literature suggests that the groups discussed here have a broader relevance in agricultural soils, although evidence in some cases is still limited. Based on available studies on the lesser-known functions of AOA, methanogens, N-DAMO and haloarchaea in C- and N-cycling, it seems that these archaeal groups and their functions mainly occur under specific conditions.

The contribution of AOA to N_2_O emissions and CO_2_ fixation seem mainly relevant under low N conditions or when N is released slowly, such as under manure fertilization. In conditions of high N availability, AOB dominate NH_3_ oxidation and are the primary contributors to N_2_O emissions. Replacing mineral fertilizers with manure can potentially favour AOA and reduce N_2_O emissions from nitrification, as AOA are suggested to produce less N_2_O per NH_3_ molecule oxidized. Shifting the balance from AOB to AOA through fertilizer treatment could therefore be a suitable strategy for lowering N_2_O emissions from agriculture soils. More specifically, management strategies can focus on shifting the AOA population to enhance the abundance of certain AOA lineages. Fertilizer application has shown to influence the abundance of AOA lineages in soil. To incorporate this knowledge into management strategies, insight is needed on the N_2_O production rates of all AOA lineages and how soil conditions such as N availability and pH affect these production rates.

More research is also needed into the CO_2_ fixation capabilities of AOA. Further studies, for example using stable isotope probing, are needed to confirm this relationship and to determine the actual rate of CO_2_ fixation of AOA in agricultural soils, as data from forest soils and marine environments suggests different fixation rates. If CO_2_ fixation by AOA significantly contributes to soil C formation, it would be promising to investigate strategies to increase the CO_2_ fixation rate of this archaeal group. Currently, the effect of manure treatments on CO_2_ fixation by AOA remains unclear, and manure addition, in the studies that are published on this topic, did not see an increase in *accA* abundance. This suggests that nutrient input might not be a suitable strategy to increase CO_2_ fixation by AOA. In this regard, setting up a field experiment to determine the effect of different fertilizers on the CO_2_ fixation rates will be required. In addition, exploring other strategies independent of nutrient input such as microbiome engineering might be a promising future direction. A recent study indicates that the CO_2_ fixation rates of bacterial autotrophs could be significantly increased through virus infection (Lu et al., 2025). No such effect has been documented for archaea yet, but this appears to be a promising approach to be explored.

Methanogens are primarily relevant in anaerobic environments. Specifically, hydrogenotrophic methanogens can contribute to CO_2_ fixation. Considering CO_2_ fixation is linked to CH_4_ production, an increase in CO_2_ fixation will also lead to an increase in CH_4_ emissions. Hence, from a sustainability perspective, methanogens are not a good target for increasing CO_2_ sequestration in agricultural soils. The same limitation is true for N_2_ fixation by methanogens, as N_2_ fixation depends on the ATP produced through methanogenesis. Low detection of N_2_-fixing methanogens through *nifH* targeted qPCR, further raises the question if methanogens are relevant for agricultural environments. Adjusting current primers or designing specific primers targeting *nifH* of N_2_-fixating archaea will help determine the significance of N_2_ fixation by this archaeal group.

Overall, due to their specific occurrence, increasing methanogen abundance is likely not an appropriate strategy for increasing C or N in most agricultural soils. Therefore, management strategies in agriculture should prioritize other approaches and focus on other CO_2_^−^- or N_2_-fixing microorganisms that provide nutrients without environmental costs. A potential alternative could be directed evolution to achieve expression of nitrogenase in crops, which might allow plants to directly carry out N transformation without relying on the soil microbiome. This approach currently has several challenges however, including the anaerobic nature of nitrogenase and potential growth defects in host cells due to high metabolic burden (Bennett et al., 2023).

Recent research revealed that a consortium of N-DAMO archaea and bacteria could be used as a promising strategy to reduce NO_3_^−^ and CH_4_ emissions in brackish ecosystems (Legierse et al., 2023). Using bioaugmentation, N-DAMO abundance reached 47–73% of the total archaeal population in sediment microcosms (Legierse et al., 2023). Implementation *in situ* has yet to be tested, however it might be a promising strategy in paddy soils or in other waterlogged environments to increase the removal of CH_4_ and NO_3_^−^ from the system.

While the abundance of N-DAMO archaea is likely low in most soils, N-DAMO growth could be stimulated through fertilization or potentially through bioaugmentation. Before N-DAMO archaea can be used for bioaugmentation in soil environments, several questions need to be answered first, including: (1) Can N-DAMO archaea survive and remain active in anaerobic soils? and (2) If so, what delivery or inoculation strategies would allow bioaugmentation without disruption of the native microbiome? Soils have different redox potentials and nutrient concentrations compared to sediments (Kalev and Toor, 2018), and survival of N-DAMO under these conditions needs to be tested. After answering these questions, small-scale microcosm and controlled field experiments are needed to determine if N-DAMO archaea meaningfully contribute to CH_4_ emission mitigation. As current studies suggest that nutrient input can be used to regulate N-DAMO, microcosm and field experiments should examine the effect of different fertilizers on their activity.

The role of haloarchaea in C- and N-cycling remains ambiguous. Notably, out of the archaeal groups reviewed here, they are the only group that has not yet been detected with a specific primer set in soils. Designing specific primers for detection in soil samples should therefore be a priority. Many primers for bacterial denitrifiers are available, some might be suitable for the detection of archaeal denitrification genes (Wei et al., 2015; Ma et al., 2019). Compared to the other archaeal groups that are reviewed here, establishing molecular detection tools will be needed to further establish the ecological significance of haloarchaea.

Future research on haloarchaea should focus on significance of their contributions to C- and N-cycling. This is both important to understand their role in natural environments and for their usage as biofertilizer. In this regard, their contributions to N_2_O formation should be the priority for future research. In saline environments, studies should focus on which microbial groups contribute most to N_2_O formation. If haloarchaea are major contributors, management strategies could be incorporated to reduce N input in saline environments. Microcosm and field experiments should be used to determine if using haloarchaea as biofertilizer increases N_2_O emissions. If this is the case, mitigation strategies are needed. This could include co-amendment with N-DAMO archaea, to reduce the amount of NO_3_^−^ in the soil. In addition, Stachler et al. (2020) showed that the endogenous CRIPSR system of haloarchaeon *Haloferax volcani* could be used to repress gene expression. This might indicate that a similar system can be used to repress genes involved in denitrification. This would reduce the risk of N_2_O emissions from haloarchaea used as biofertilizer.

In summary, archaea have diverse functional potential in agriculture soils, but the significance of their roles seems context dependent. Further development of molecular detection tools, specifically for haloarchaea, and culture dependent tools, such as culturing approaches and tracing studies can bridge knowledge gaps. Steering the composition of the soil microbiome might be possible through changes in nutrient management, such as application of organic fertilizer instead of chemical fertilizer, or through microbiome engineering. To harness the potential of N-DAMO archaea for the removal of CH_4_ and NO_3_^−^, bioaugmentation might be a suitable strategy. Nutrient cycling depends on contributions of the entire soil microbiome, therefore the specific context of the interactions between different groups of organisms in the microbiome should be further understood to fully leverage the functions of archaea in C- and N-cycling. This can then lead to improved nutrient management strategies in agriculture.
